# Assessment of the Characteristics of Orientation Distribution Functions in HARDI Using Morphological Metrics

**DOI:** 10.1371/journal.pone.0150161

**Published:** 2016-02-26

**Authors:** Chang-yu Sun, Yue-min Zhu, Chun-yu Chu, Feng Yang, Wan-yu Liu, Julie R. Korenberg, Edward W. Hsu

**Affiliations:** 1 CREATIS, CNRS UMR 5220, Inserm U1044, INSA Lyon, University of Lyon, Villeurbanne, France; 2 Harbin Institute of Technology, Harbin, China; 3 School of Computer and Information Technology, Beijing JiaoTong University, Beijing, China; 4 Department of Pediatrics, University of Utah, Salt Lake City, Utah, United States of America; 5 Department of Bioengineering, University of Utah, Salt Lake City, Utah, United States of America; University of Ulm, GERMANY

## Abstract

Orientation distribution functions (ODFs) are widely used to resolve fiber crossing problems in high angular resolution diffusion imaging (HARDI). The characteristics of the ODFs are often assessed using a visual criterion, although the use of objective criteria is also reported, which are directly borrowed from classic signal and image processing theory because they are intuitive and simple to compute. However, they are not always pertinent for the characterization of ODFs. We propose a more general paradigm for assessing the characteristics of ODFs. The idea consists in regarding an ODF as a three-dimensional (3D) point cloud, projecting the 3D point cloud onto an angle-distance map, constructing an angle-distance matrix, and calculating metrics such as length ratio, separability, and uncertainty. The results from both simulated and real data show that the proposed metrics allow for the assessment of the characteristics of ODFs in a quantitative and relatively complete manner.

## Introduction

The orientation distribution function (ODF) [1] is a quantity used to describe the orientation architecture of the tissue’s fibers or fiber bundles; it gives the probability of diffusion in different directions. ODF is often estimated or reconstructed from high angular resolution diffusion imaging (HARDI) such as q-ball imaging (QBI) [2] using spherical sampling. In this field, most existing works put emphasis on improving the quality of ODF using normalization and regularization [2,3], change of basis [4–6], sharping deconvolution [7], compressed sensing [8], etc. Other quantities have also been used to describe fiber orientation or crossing, including the fiber orientation distribution (FOD) from the spherical deconvolution method [9,10], the orientation map derived from the diffusion orientation transform (DOT) based on the Fourier transform relationship between water displacement probability and diffusion-attenuated magnetic resonance (MR) signal expressed in spherical coordinates [11], and the water molecule displacement probability function [12] using the mixture of Wisharts.

In the past, a number of metrics have been reported to assess the quality of quantities in HARDI. The most important metric is the minimum resolvable fiber crossing angle or angular resolution. Other angular metrics include the angular error [3,5,8,9,13], percentage of success [3,9,13], angular average and standard deviation errors [3,9], and probability of false crossing fiber detection [13,14]. These metrics only encode fiber detection or angular information [3]. In addition to basic angular information, the anisotropic characteristic of HARDI quantities was also reported. We can cite the generalized fractional anisotropy (GFA) metric that calculates the ratio of the standard deviation to the root mean square of ODF values [2], and the generalized anisotropy (GA) [15] that quantifies the anisotropy from higher-rank diffusion tensors in teams of the variance of the diffusivities. These metrics present the particularity of summarizing the shape of the ODF into a single anisotropy measure, and much of information inherent in HARDI data is discarded.

Other metrics encode more global shape information of ODFs, such as the mean square error [3], root-mean square error (RMSE) [5], and normalized mean squared error (NMSE) [8]. In particular, in [16], a global description of the ODF, called the Kullback–Leibler (sKL) metric originating from information theory, was proposed that uses gold standard ODFs as ground truth to assess how accurately the diffusion profile could be reconstructed from sub-sampled data based on different angular sampling schemes. The sKL metric allows for the measurement of the discrepancy between the reconstructed and ground-truth ODFs. These metrics can be used to compare ground-truth ODFs and estimated ODFs. However, in real-data cases, the ground truth is difficult to obtain. In [17], the authors used an objective metric from statistics, called the Dip test [18] that estimates the maximum distance between the empirical distribution function and the closest unimodal distribution function, to compare the quality of crossing fibers in various HARDI quantities such as ODFs, FODs, orientation maps, and water molecule displacement probability functions. Like the sKL metric, the Dip test metric can quantify the quality of ODFs (or other HARDI quantities) showing the same fiber crossing. But, unlike the sKL metric, the Dip test metric does not require ground-truth ODFs. However, it requires sampling a direction from the ODFs to be able to perform the Dip test in a one- or two-dimensional (2D) space, and such sampling should be appropriate.

In addition to the basic angular information, anisotropic information, and global shape information, other characteristics of ODFs were also addressed in the literature, which consist in analyzing the peaks of ODFs, such as the uncertainty and volume fraction of the peaks of ODFs. In [19], the peak anisotropy was defined as the variance of the Hessian eigenvalues, in a similar way to the fractional anisotropy of the diffusion tensor, to reflect local fiber bending or fanning. In [20], the uncertainty of ODFs was measured using bootstrap analysis involving the resampling of originally acquired diffusion-weighted datasets. In [21], a rotation invariant feature that takes the eigenvalues of spherical functions as rotation invariant metrics was used to describe the shape of ODFs, which implies that the metric was directly dependent on the spherical harmonic representation of HARDI signals used.

**Fig 1:**
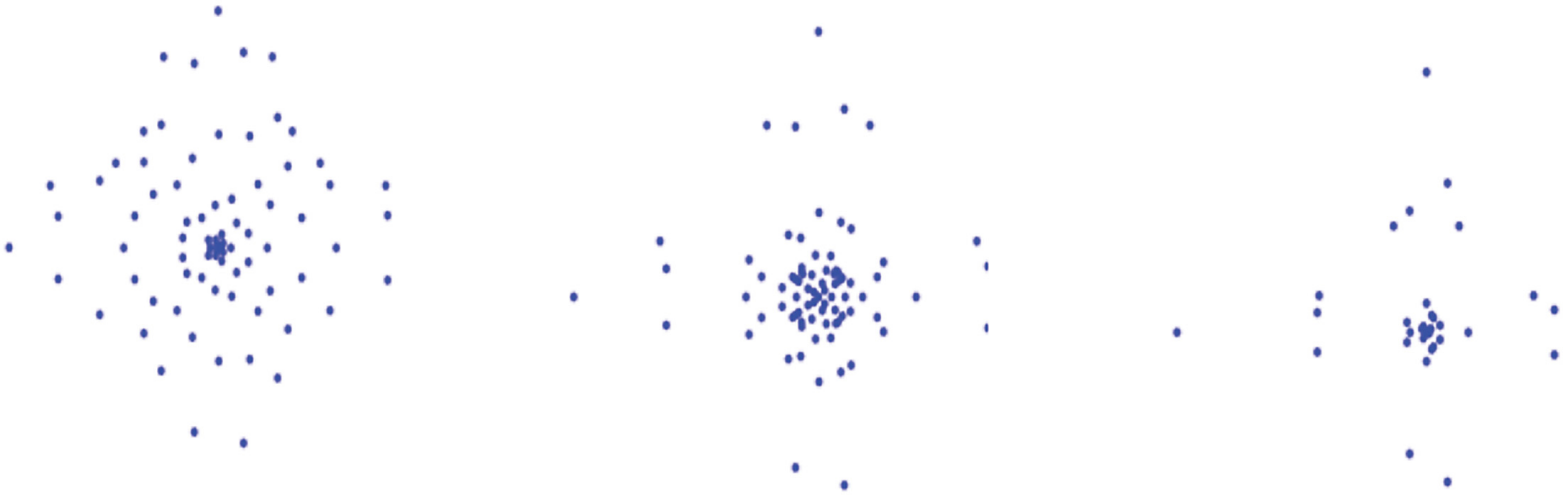
Three different examples of 3D point cloud representation.

The aforementioned HARDI quantities share a common point, namely, they are all composed of *N* samplings of the sphere (*N* 3D points), where the direction of each sampling corresponds to one reconstruction direction of HARDI and the distance to the origin of each sampling corresponds to the probability density function of water diffusion along that reconstruction direction [22,23]. This led us to regard a HARDI quantity as a three-dimensional (3D) point cloud (Fig 1), in which each point corresponds to a vector originating from the coordination system origin.

The direction and the length of the vector designate the direction and the amount of the diffusion, respectively. Seen from this point of view, the ODFs (we observe visually) are in fact the 3D surface representations of 3D point clouds. Also for this reason, for simplicity, in what follows, ODF and 3D point cloud will be used interchangeably, although they have different meanings. Then, the next step would be to try to gain insights into the spatial structure and shape of the 3D point cloud in a general and systematic manner. To do this, we project the 3D point cloud in an angle-distance plane to obtain a 2D angle-distance map (ADM), construct an angle-distance matrix (ADMAT), and calculate the metrics such as the length ratio, separability, and uncertainty.

## 3D Point Cloud Characteristic Assessment Paradigm

**Fig 2:**
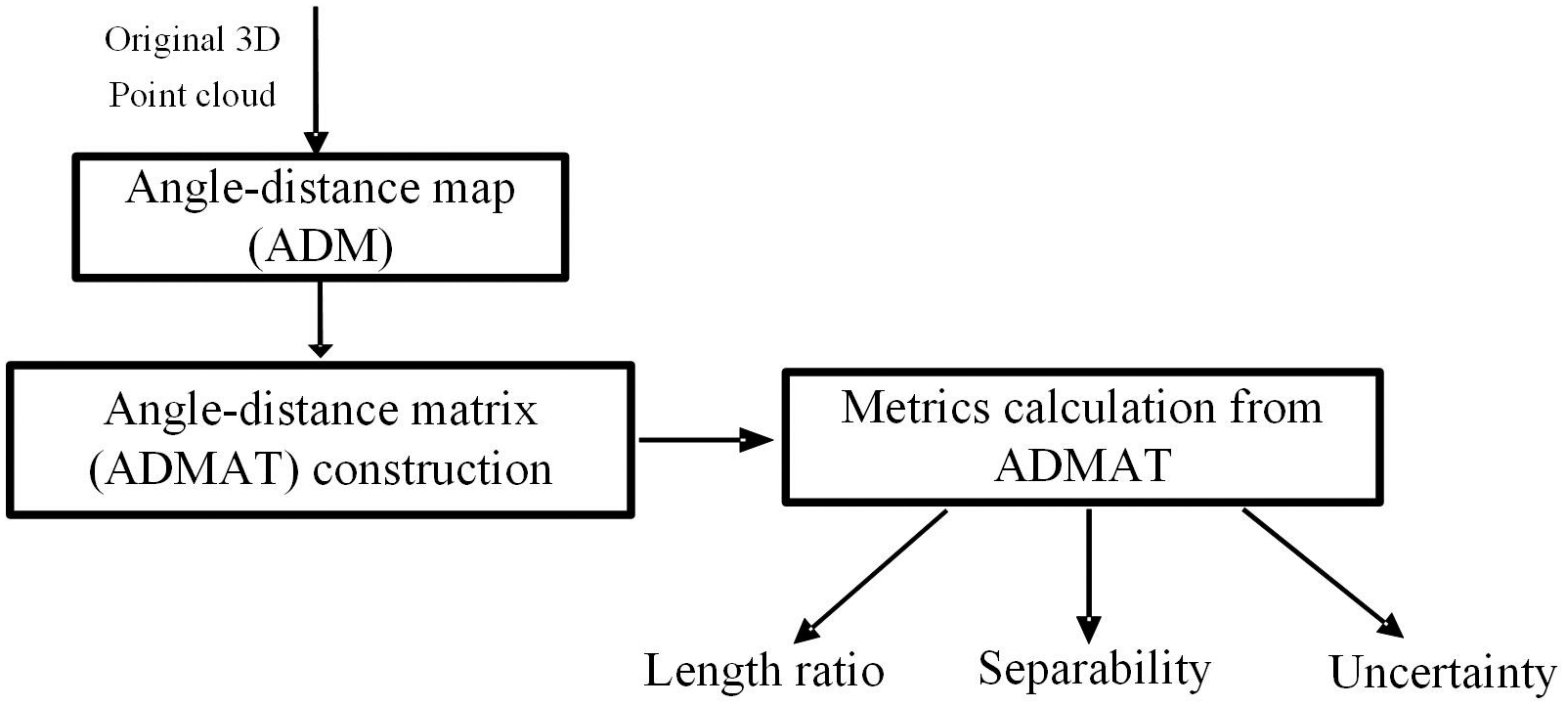
Flow chart for calculating morphological measures.

The flow chart for calculating the proposed morphological metrics is illustrated in Fig 2.

### Construction of the Angle-Distance Map (ADM)

**Fig 3:**
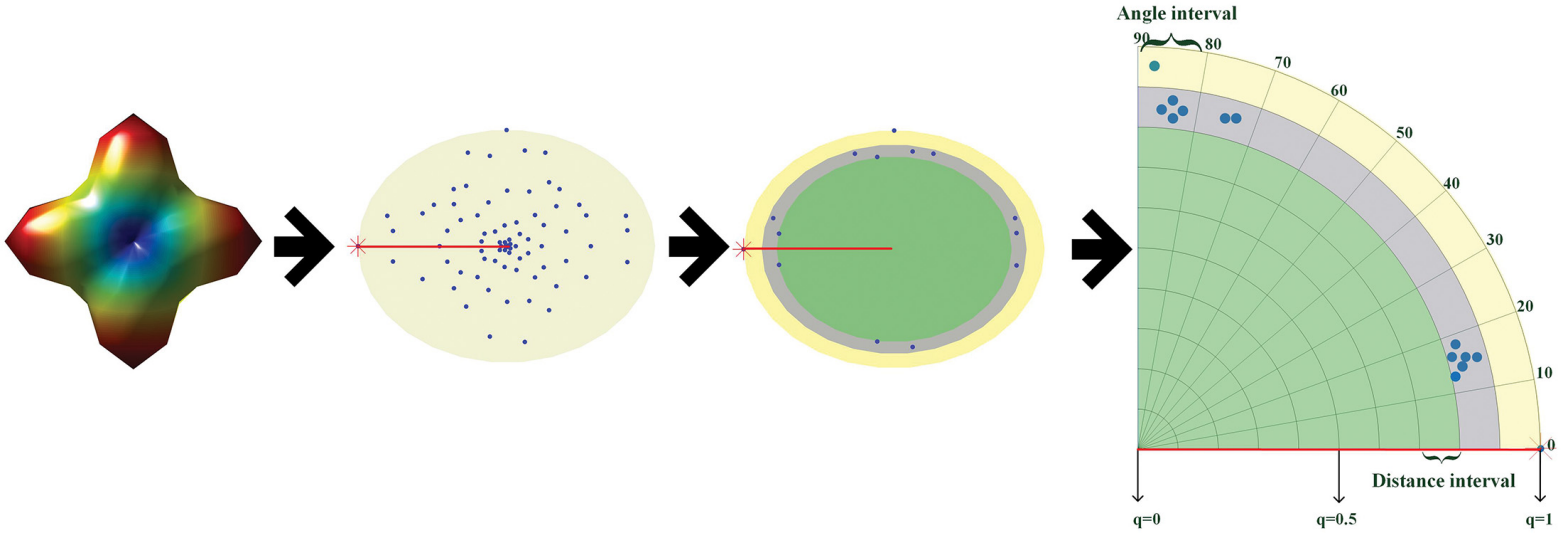
Projection of 3D points on the angle-distance map. From left to right: ODF, 3D point cloud with the red point indicating the reference vector, illustration of 3D points in two annuluses, and projection of the 3D points on the ADM.

The 3D surface representation of ODFs was addressed in [1–3], with a variant reported in [17]. It is often expressed in spherical coordinates. To better visualize and handle ODFs or more generally 3D point clouds, we express them in Cartesian coordinates by $\vec{q}={\left(x,y,z\right)}^{T}=q\vec{e}$. The 3D point cloud can then be described by a set of vectors. Since a vector is defined by radial distance and orientation, we introduce these two parameters to characterize the 3D point clouds. If *q*_*m*_ represents the maximal distance with ${\vec{e}}_{m}$ as its direction, we then take ${\vec{q}}_{m}$ as a reference vector. From the reference vector, we construct an ADM formed of small areas delineated by radial lines spaced by 90° / *N*_*a*_ with $N_{a}\in \mathbb{N}$, and circles of radius *k* / *N*_*c*_ with k = 1,…,*N*_*c*_ and $N_{c}\in \mathbb{N}$, where *N*_*c*_ designates the number of partitions in the radial direction and *N*_*a*_ the number of partitions of the angle range. A small area is then the intersection of an annulus and a fan sector. The annulus is determined by (*i*−1) / *N*_*c*_ and *i* / *N*_*c*_, and the fan sector by 90(*j*−1) / *N*_*a*_ and 90*j* / *N*_*a*_. The number of small areas is determined by the choice of *N*_*a*_ and *N*_*c*_. We now project the given vectors onto this ADM. To do this, we first calculate the angle between each vector $\vec{q}$ and the reference vector ${\vec{q}}_{m}$ (here we calculated the line angles, thus the angles are between 0 and 90°). Since the distance *q* of $\vec{q}$ to the origin is known (equal to its length), we have the values of the angle and distance, which enables us to put the vectors in the corresponding area of the ADM (Fig 3). If the maximum distance appears in two or more directions, we can choose any of them as the reference direction.

**Fig 4:**
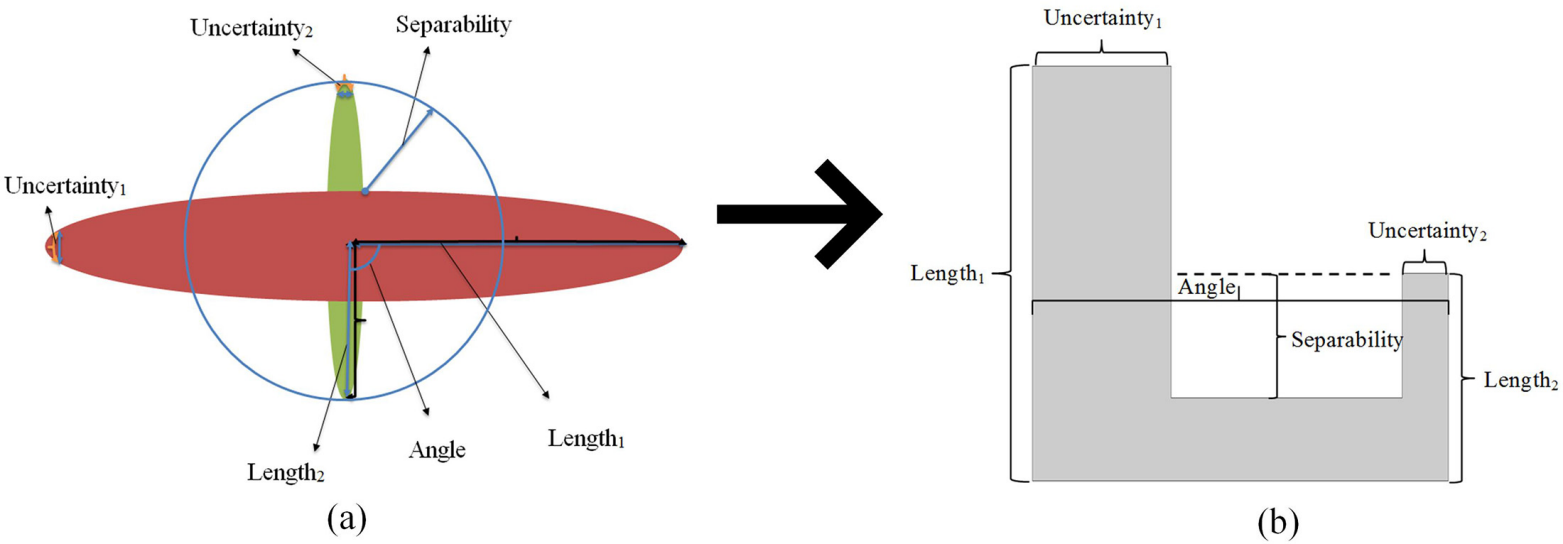
Illustration of the definition of the length ratio, separability, and uncertainty metrics in the 3D point cloud (a) and in the schematized ADMAT (b).

Once all the ODF points are projected on the ADM, we analyze and characterize the distribution of the projections. To this end, we first define three metrics (whose mathematical expressions will be given in next section): The length ratio that describes the main direction diffusivity, the separability that reflects the 3D point cloud’s ability to separate main directions, and the uncertainty that indicates the width of the 3D point cloud’s tine or peak. The definition of these metrics is illustrated in Fig 4(a). The values of the length ratio range from 0.1 to 0.9, those of the separability from 0 to 1, and those of the uncertainty from 0 to 1.57(90*π* / 180). The greater the length ratio, the closer the lengths between the two fibers. The greater the separability, the more the fibers can be easily separated. The smaller the uncertainty, the thinner the peaks of the 3D point cloud.

To calculate these metrics, we will construct in the following the angle-distance matrix (ADMAT).

### Construction of the Angle-Distance Matrix (ADMAT)

In Fig 4(b) we illustrate an ADMAT and its relationship with the 3D point cloud. We represent the 3D point cloud in Fig 4(a) in a schematized 2D ADMAT (the details will be given in next section) in Fig 4(b), where the schematized ADMAT presents two rectangular bars. The distance between the two bars represents to some extent the crossing angle. Then, from the 2D map obtained, we calculate the three metrics directly. The length ratio is defined as the ratio of shorter bar length to longer bar length using min(Length_1_,Length_2_)/max(Length_1_,Length_2_), the separability as the depth from the bottom to the top of the shorter bar, and the uncertainty as the sum of the widths of the two bars divided by 2.

To construct the ADMAT, we now determine how a 3D point is projected in the corresponding small area of the ADM. Since the crossing angle ranges from 0 to 90°, a 3D point is then projected in the corresponding annulus according to

$$d(i)=\left\{\vec{q}|\left(i-1\right)/N_{c}\leq q/q_{m}\leq i/N_{c}\right\},$$ (1)

which gives the set *d*(*i*) of the 3D points falling inside the *i*^*th*^ annulus.

At the same time, we consider the projection of the same 3D point in the fan sector using

$$ang\left(j\right)=\left\{\vec{q}|90(j-1)/N_{a}\leq arccos({\vec{q}}_{m}\cdot \vec{q}/q_{m}q)\leq 90j/N_{a}\right\},$$ (2)

which gives the set *ang*(*j*) of the 3D points falling inside the *j*^*th*^ fan sector.

Thus, the set *angd*(*i*, *j*) of 3D points falling into the small area (*i*, *j*) of the ADM is given by

$$angd\left(i,j\right)=\left\{\vec{q}|\begin{array}{l} \left(i-1\right)/N_{c}\leq q/q_{m}\leq i/N_{c},\\ 90(j-1)/N_{a}\leq arccos({\vec{q}}_{m}\cdot \vec{q}/q_{m}q)\leq 90j/N_{a} \end{array}\right\}.$$ (3)

We count the number of the projections of 3D points in the area (*i*, *j*) using *N*(*i*,*j*) = *Card*(*angd*(*i*, *j*)), where *Card*() designates the cardinal number. We then construct the ADMAT by calculating each of the elements in the area as

$$M_{i,j}=\sum_{p=1}^{i}c_{p}N\left(p,j\right),$$ (4)

where *c*_*p*_ denotes a coefficient that weights the influence of distance on the shape of a 3D point cloud. Because of accumulation effect in Eq (4), the last line of ADMAT always has the greatest value.

Eq (4) expresses a general idea of weighting cloud points according to their distance from the origin. Since the points near the origin do not contribute much to the shape of the 3D point cloud, we will attenuate their influence using

$$M_{i,j}=\sum_{p=1}^{i}a^{-(p-1)}N\left(p,j\right),$$ (5)

where *a* > 1 is a constant. In the present study, we chose *a* = 10.

**Fig 5:**
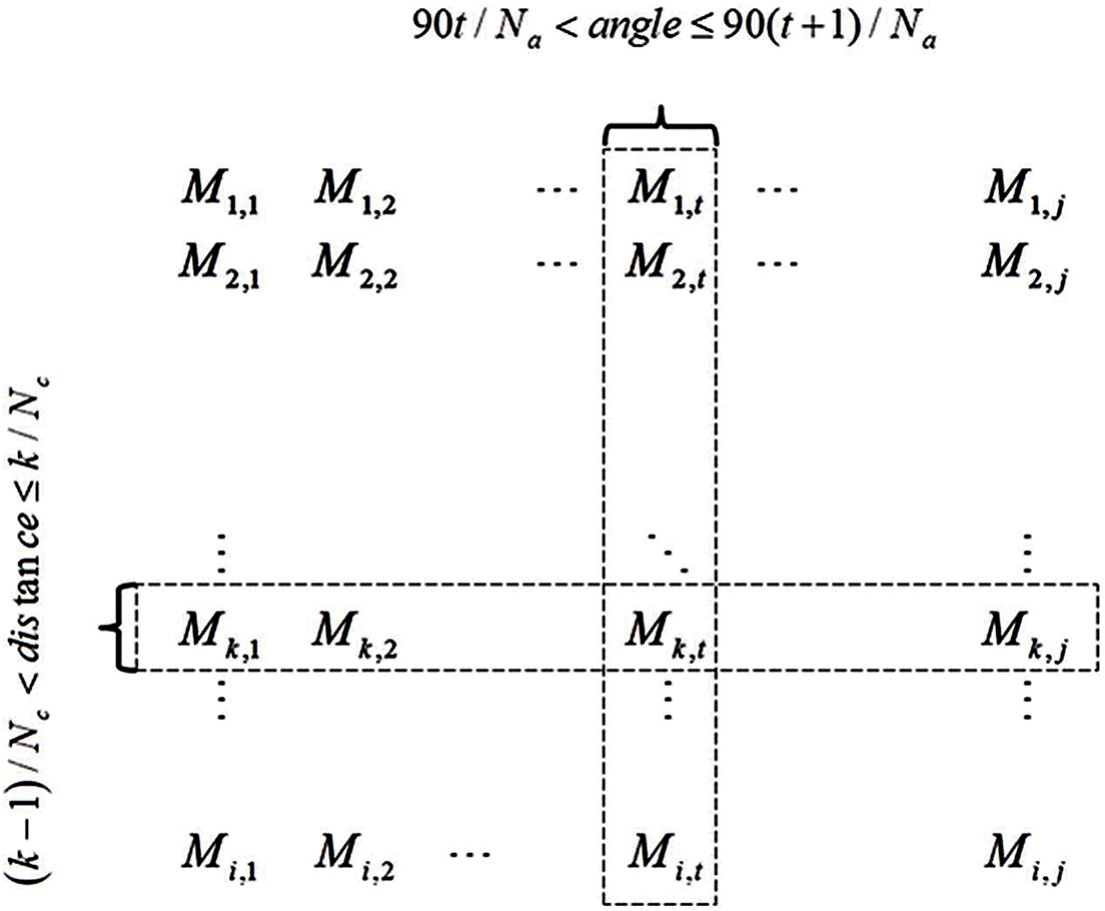
Definition of the ADMAT.

In ADMAT, the column number and the row number indicate the angular interval and the distance interval, respectively (Fig 5). Thus, ADMAT encodes the distance and angle information of 3D point clouds.

**Fig 6:**
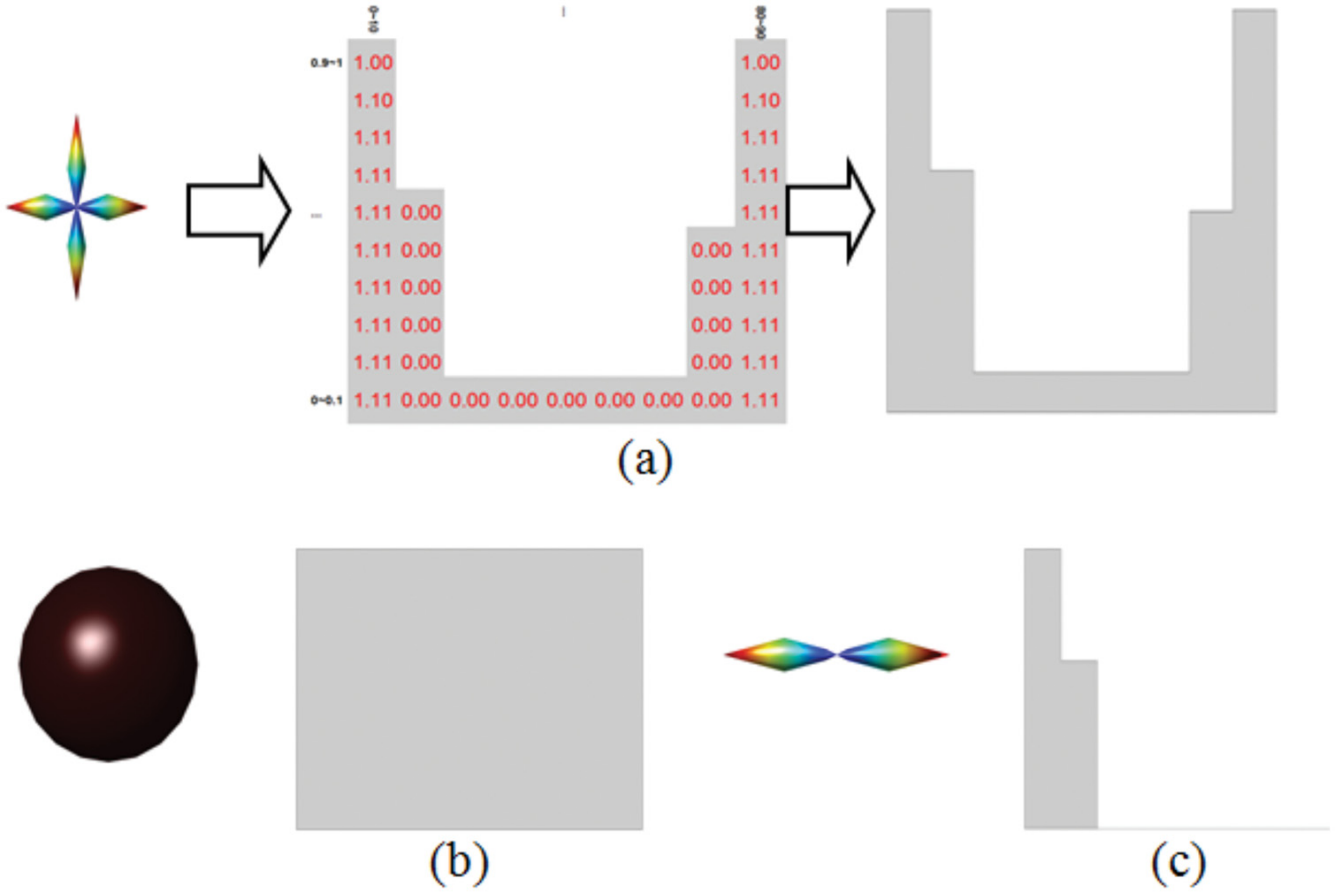
3D point cloud and schematized ADMAT of (a) 90° crossing fiber, (b) free diffusion, and (c) single fiber.

In Fig 6 we give some typical examples of ODFs visualized as 3D color surface, the corresponding ADMAT, and the schematized ADMAT. For the anisotropic orthogonal fiber crossing cloud in Fig 6(a), ADMAT presents two regions separated by a white region, and full-zero columns or columns having very small values between two columns. For the spherical isotropic cloud shown in Fig 6(b), which represents a free diffusion situation, each element of the schematized ADMAT appears as gray, there are no white regions, and the whole ADMAT is gray. For the anisotropic fiber cloud shown in Fig 6(c), ADMAT presents a gray region near its first column, and the other regions are white.

**Fig 7:**
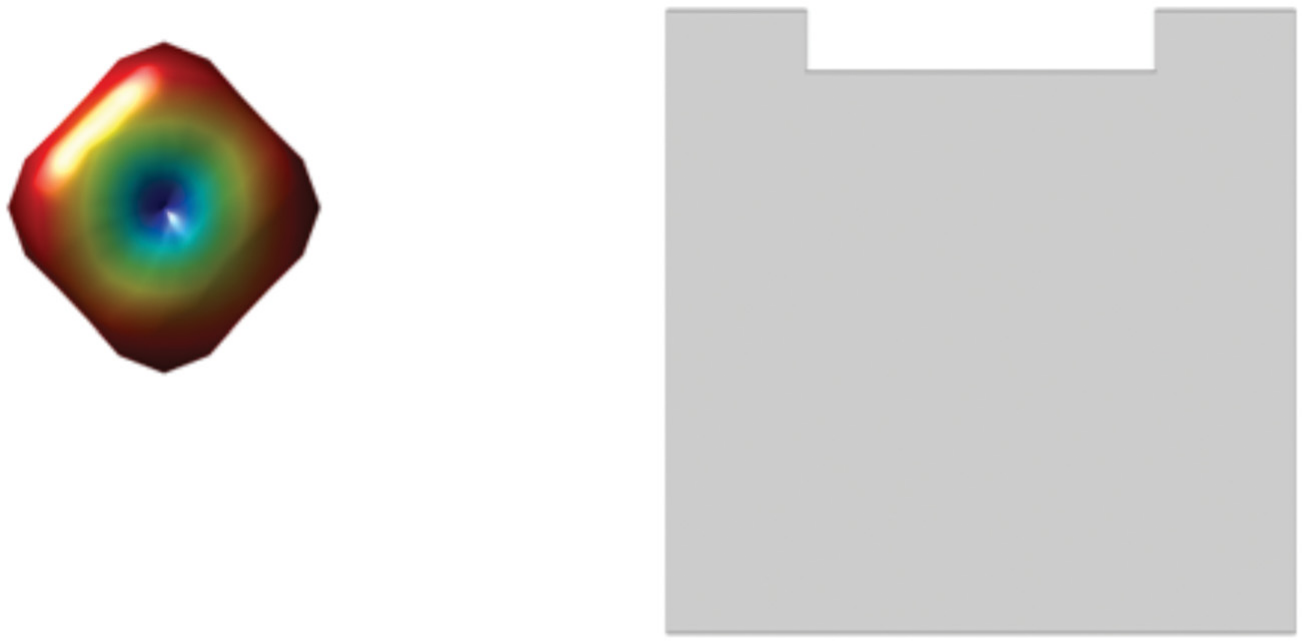
Example of a fiber crossing that is difficult to assess with 3D color surface representation (left), but much easier to see with the schematized ADMAT (right).

It is to be underlined that ADMAT encodes richer information than 3D surface representation does. Fig 7 aims to illustrate this fact, in which the fiber crossing is difficult to assess in the 3D color surface representation of the point clouds, but it is clearly indicated by the schematized ADMAT due to the presence of a white region in the middle of the first line; the schematized ADMAT indicates that the separability is very small in this ODF and the uncertainty of two fiber crossing is very large.

### Calculation of Morphological Criteria from ADMAT

From the ADMAT, we define two sets representing, respectively, the positions *m*_*k*,*x*_ of zeros and the positions *n*_*k*,*x*_ of non-zeros in the *k*^th^ line of ADMAT

$$\{\begin{array}{c} m_{k,x}=\left\{j|i=k,M_{i,j}=0\right\}=\left\{m_{k,1},m_{k,2},m_{k,3}\ldots m_{k,l}\right\}\\ n_{k,x}=\left\{j|i=k,M_{i,j}\neq 0\right\}=\left\{n_{k,1},n_{k,2},n_{k,3}\ldots n_{k,g}\right\} \end{array},\;1\leq x\leq N_{a}$$ (6)

with 1 ≤ *k* ≤ *N*_*c*_ and *Card*(*m*_*k*,*x*_) + *Card*(*n*_*k*,*x*_) = *l* + *g* = *N*_*a*_.

We then, for the given *k*^th^ line, calculate the finite difference of *m*_*k*,*x*_ and *n*_*k*,*x*_ to detect discontinuous positions

$$\begin{array}{c} r_{k,p}=\left\{x|m_{k,x+1}-m_{k,x}\neq 1\right\}=\left\{r_{k,1},r_{k,2},r_{k,3}\ldots r_{k,ls}\right\}\\ v_{k,p}=\left\{x|n_{k,x+1}-n_{k,x}\neq 1\right\}=\left\{v_{k,1},v_{k,2},v_{k,3}\ldots v_{k,gs}\right\} \end{array},1\leq p\leq N_{a}$$ (7)

where *ls* and *gs* designate the number of discontinuities in *m*_*k*,*x*_ and *n*_*k*,*x*_, respectively. *r*_*k*,*p*_ and *v*_*k*,*p*_ represent the set of discontinuities in *m*_*k*,*x*_ and *n*_*k*,*x*_, respectively.

If at the *k*^th^ line *v*_*k*,*p*_ = ∅, this means that either the 3D point cloud contains only one main direction or it cannot distinguish different directions. Otherwise, at the *k*^th^ line, the 3D point cloud contains *Card*(*v*_*k*_) main directions. For example, in case there are two directions, we can determine the number of lines (*μ* × *N*_*c*_) at which two directions can be resolved

$$\mu =\left\{k/N_{c}|v_{k,p}\neq \emptyset \right\}=\left\{\mu_{1},\mu_{2},\mu_{3}\ldots \mu_{ns}|ns\leq N_{c}\right\},$$ (8)

where *ns* is the number of lines whose *v*_*k*,*p*_ is not empty.

We then define an operator *fset*() that takes a discontinuity as input and outputs a set of positions of zeros or non-zeros in the *k*^th^ line of the ADMAT

$$fset(r_{k,p})=\left\{ \begin{array}{l} \left\{m_{k,1},m_{k,2},m_{k,3}\ldots m_{k,r_{k,1}}\right\},\;p=1\\ \left\{m_{k,r_{k,p-1}}+1,m_{k,r_{k,p-1}+1},m_{k,r_{k,p-1}+2}\ldots m_{k,r_{k,p}}\right\},\;p=2,\ldots ,ls \end{array}\right.$$ (9)

In the same manner, we can get the set *fset*(*v*_*k*_) of positions having non-zero value.

The width of the *lw*^th^ main direction at (*μ*_1_ × *N*_*c*_)^th^ line is $\pi Nv_{\mu_{1},lw}/2N_{a}$ with $Card(fset(v_{\mu_{1}\times N_{c},lw}))=Nv_{\mu_{1}\times N_{c},lw}$; *μ*_1_ × *N*_*c*_ is the line number of ADMAT at which fiber crossing is resolved for the first time. In this case, we can further determine how well the two directions can be resolved. To do this, we now compute the aforementioned morphological metrics: length ratio (1−*μ*_1_), separability (*μ*_ns_ − *μ*_1_) + 1 / *N*_*c*_, and uncertainty $\pi (Nv_{\mu_{1}\times N_{a},1}+Nv_{\mu_{1}\times N_{a},2})/4N_{a}$. The algorithm is given here:

Input: *Q($\vec{q}$)* (3D point cloud)

Outputs: length ratio, separability and uncertainty metrics

*M*_*Na*,*Nc*_ ← *Nc, Na, Q($\vec{q}$)*

For *i = 1 to Nc* do

 For *j = 1 to Na* do

 {*m*_*i*,*x*_} ← find the positions of zeros in {*M*_*i*,*j*_}

 {*n*_*i*,*x*_} ← find the positions of no zeros in {*M*_*i*,*j*_}

 End For

 If *n*_*i*,*x+1*_*-n*_*i,x ≠*_*1*

 { *v*_*i*,*p*_ } ← *x*

 End if

 Calculate the sets of zeros and non-zeros using *fset*()

End For

 if {*ν*_*i*,*p*_} ≠ ∅

 {*μ*} ← {*i*}*/Nc*

 End if

separability ← *μ*_*ns*_ − *μ*_1_ + 1 / *N*_*c*_

length ratio of 2-fiber system ← (1−*μ*_1_)

uncertainty of *lw*^th^ main direction at (*μ*_1_ × *N*_*c*_)^*th*^ line ← $Card(fset(v_{\mu_{1,lw}}))/2N_{a}$uncertainty of 2-fiber system at (*μ*_1_ × *N*_*c*_)^*th*^ line ← $\pi (Nv_{\mu_{1}\times N_{c},1}+Nv_{\mu_{1}\times N_{c},2})/4N_{a}$

**Fig 8:**
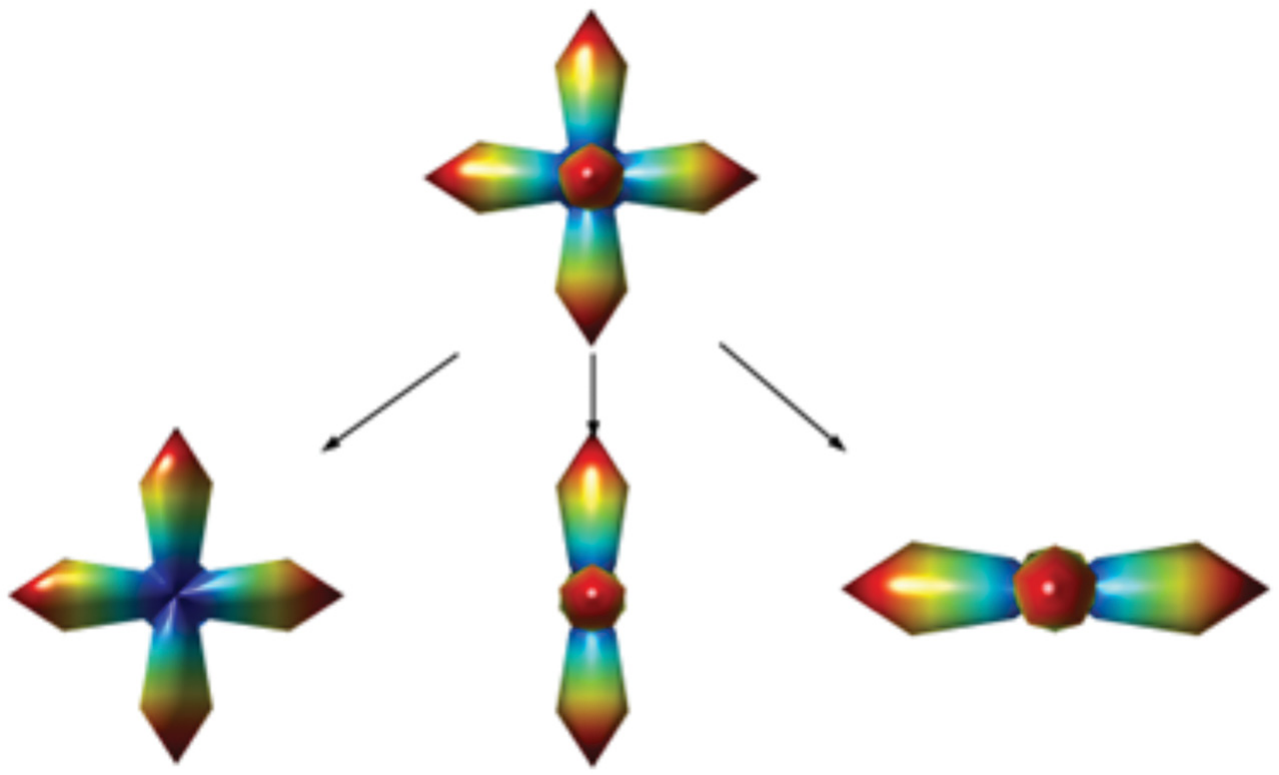
Example of three-fiber crossing that is paired off, and the measurement method of two-fiber crossing that can be applied to each pair to calculate the length ratio, separability, and uncertainty.

In the case of a three-fiber crossing, we divide the 3D point cloud into three maps of 3D points; each map contains two local maximum directions, and the local maximum directions of the estimated ODF and the neighboring 3D points around local maxima are determined by the method proposed in [24]. We then apply the two-fiber system method shown in the algorithm to analyze each pair (Fig 8). If there are more than two fibers in a voxel, we will sum the values of all the two-by-two length ratios or separabilities and sum the uncertainties of all the fibers, as shown in Fig 8.

## Experiments and Results

To assess the effectiveness of the proposed metrics (length ratio, separability, and uncertainty), both simulated and real 3D point clouds were used. In the case of simulated data, both noise-free and noisy (Rician noise, with different signal-to-noise ratios) data were generated. The ODFs presenting different crossing angles (for two-fiber systems: 90°, 70°, 50°, 30° and for three-fiber systems: 90°) were considered. The proposed metrics were also compared with existing metrics such as MSE, sKL, RMSE, and NMSE.

The real data come from a fixed and excised macaque brain hemisphere. The laboratory staff provided for the macaque, at the least, 3 daily visits of 15 minutes or more. Visit activities included snacks, singing to monkey, reading to monkey, etc. The macaque was fed a commercial diet from Harlan and foraging feeds from Bio-Serv. The environmental enrichment was provided in accordance with the standard operating procedures, including foraging, positive interaction with caretakers, varied food items. The subject was euthanized by giving an overdose of thiopental exsanguinated via cardiac puncture, and perfused using 4% paraformaldehyde prior to well-established protocols. The entire study was approved by the Institutional Animal Care and Use Committee of University of Utah. The data were acquired on a Bruker 7T scanner [25]. The acquisition parameters are the following: Echo time (TE) = 39ms, repetition time (TR) = 500ms, number of excitations (NEX) = 1, voxel size = 0.5×0.5×0.5 mm^3^, number of slices = 70, and matrix size = 100x75. The diffusion encoding was performed in 96 directions with a b-value of 5,000 s/mm^2^.

### Simulation Results

The proposed morphological metrics have been tested on different ODFs corresponding to different configurations of one fiber, two fibers, or three fibers.

The diffusion signal was simulated using the multi-tensor model $S\left({\vec{g}}_{i}\right)=\Sigma_{k}P_{k}\text{exp}(-b{\vec{g}}_{i}{}^{T}D_{k}{\vec{g}}_{i})$, where *P*_*k*_ is the apparent volume fraction of the voxel with diffusion tensor *D*_*k*_, *b* the diffusion sensitization factor, and ${\vec{g}}_{i}$ the direction of the diffusion gradient [26]. To generate different crossing angles, we rotated the following diffusion tensor [27]:

$$D_{1}=\left[\begin{array}{ccc} 1.7\times 10^{-3} & 0 & 0\\ 0 & 0.3\times 10^{-3} & 0\\ 0 & 0 & 0.3\times 10^{-3} \end{array}\right]\;mm^{2}/s,$$

whose FA = 0.81, MD = 0.76 × 10^−3^ s/mm^2^, and *P*_1_ = *P*_2_ = 0.5. In the present simulation study, we defined *N*_*a*_ = 9, *N*_*c*_ = 10, and *c*_*p*_ = 10^−(i−1)^.

In the simulation, Rician noise was used. The signal-to-noise ratio (SNR) is defined as

$$SNR=20\times {\text{log}}_{10}\left(\sqrt{\frac{\sum_{k=1}^{K}{\left(S_{k}\right)}^{2}}{\sum_{k=1}^{K}{\left(S_{k}-SN_{k}\right)}^{2}}}\right),$$ (10)

where *S*_*k*_ represents noise-free signal and *SN*_*k*_ the signal corrupted by Rician noise.

**Fig 9:**
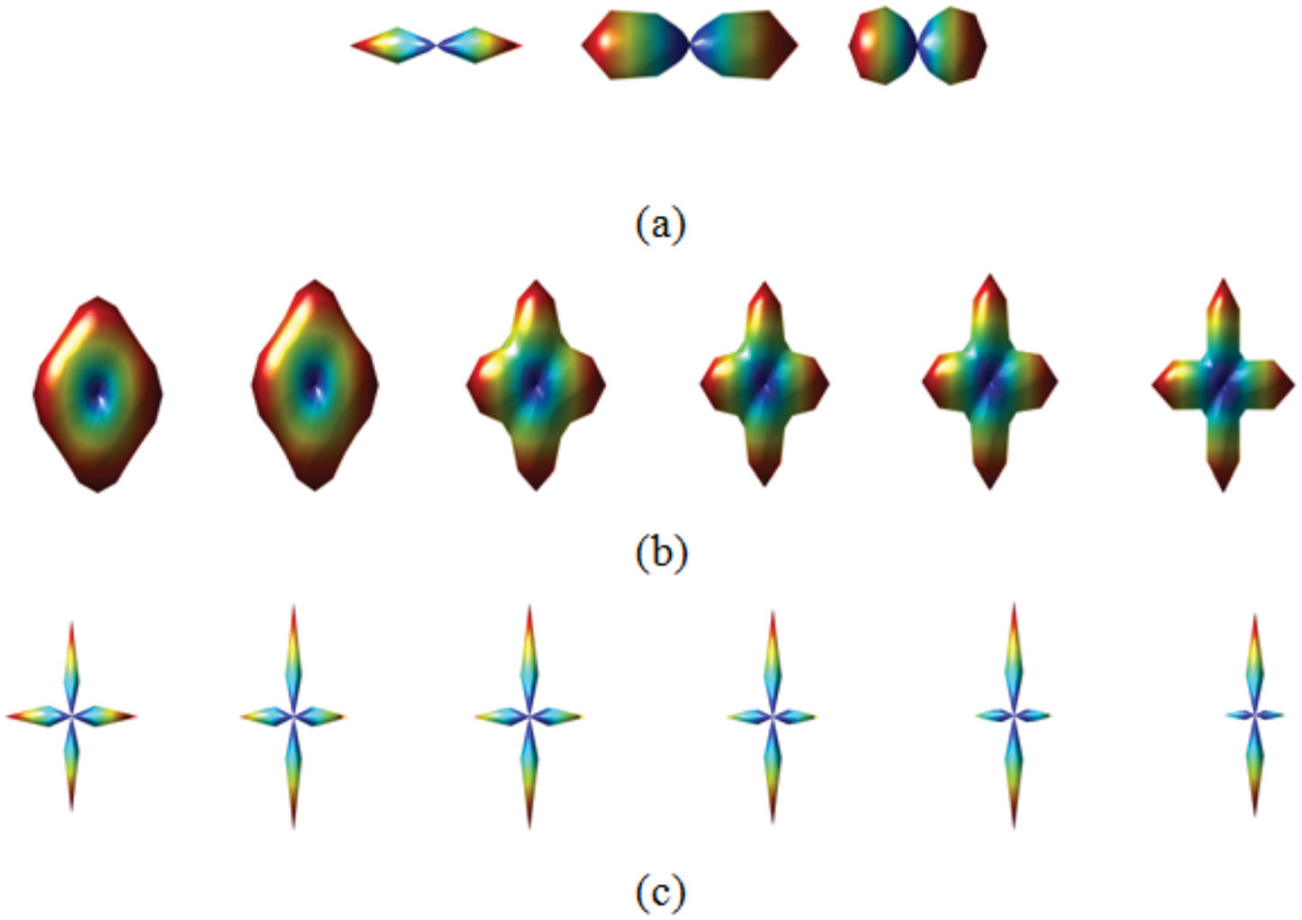
(a) Three ODFs presenting different uncertainties. (b) Six ODFs presenting different separabilities. (c) Six ODFs presenting different length ratios.

In Fig 9(a), three 3D point clouds presenting a single main direction but different uncertainties are shown. In Fig 9(b) and 9(c), we show different 3D point clouds that all have the same angle of maximum directions. Visually, the 3D point clouds corresponding to fiber crossing are not the same. Table 1 gives the quantitative measurements of this visual difference.

**Table 1:** Measurements of the uncertainty of the ODFs in Fig 9(a), the separability of the ODFs in Fig 9(b), and the length ratio of the ODFs in Fig 9(c).

	ODF 1	ODF 2	ODF 3	ODF 4	ODF 5	ODF 6
**Uncertainty in** Fig 9(a)	0.17	0.34	0.52	-	-	-
**Separability in** Fig 9(b)	0.1	0.2	0.3	0.4	0.5	0.6
**Length ratio in** Fig 9(c)	0.9	0.8	0.7	0.6	0.5	0.4

Fig 9(b) shows the separability levels from 0.1 to 0.6 and Fig 9(c) the different length ratio levels.

**Fig 10:**
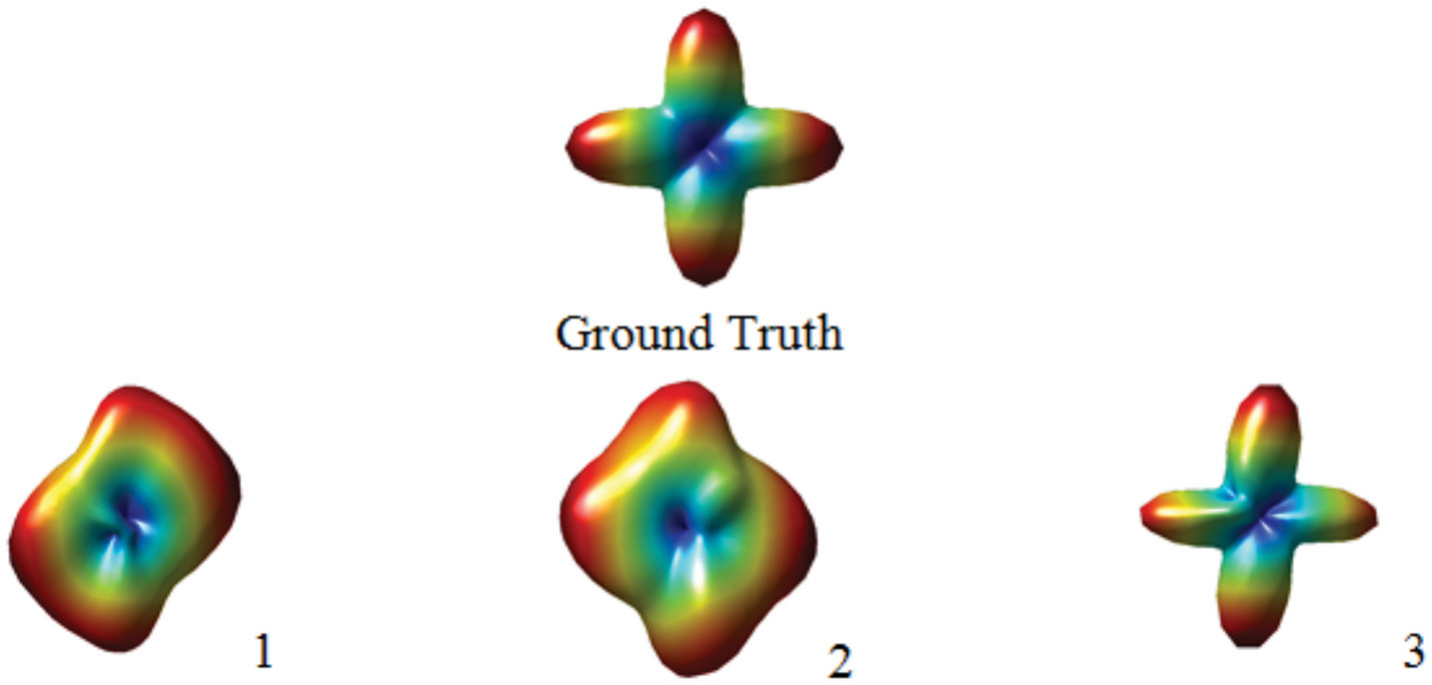
Comparison of three noisy ODFs (bottom row) and their noise-free version (ground truth).

Fig 10 shows the ground truth ODF of 90° crossing and its three different noisy versions obtained by adding Rician noise to the original diffusion signals. They are compared using MSE, sKL, RMSE, NMSE, and the proposed morphological metrics. Visually, ODF 3 has the best quality ODF, because it shows the clearest crossing among these the three ODFs and is closer to the ground truth. ODF 2 is a little better than ODF 1, because it shows clearer crossing than ODF 1. More quantitatively, each of the metrics MSE, sKL, RMSE, and NMSE gives different results for the three noisy versions of the same noise-free ODF: MSE x 10^7^ (from ODF 1 to ODF 3, the values are 3.4, 3.5, and 4.1, respectively), RMSE x 10^4^ (5.8, 5.9, and 6.4), and NMSE (0.034, 0.037, and 0.16). According to these values, ODF 1 is the closest to the ground truth, and ODF 2 is closer to the ground truth than ODF 3. With sKL (0.11, 0.1 and 0.16), ODF 2 is the closest to the ground truth. Clearly, these results are not consistent with the obvious visual observation. On the contrary, the proposed metrics always give consistent results with the visual observation: length ratio (0, 0.8, 0.85), separability (0, 0.05, 0.30), and uncertainty (1.57, 1.22, 0.87).

For a given fiber configuration, we generated three types of ODFs, called Field 1 [3] obtained by analytical q-ball imaging (AQBI), Field 2 obtained by q-ball imaging in constant solid angle (CSA) [17], and Field 3 obtained by constrained spherical deconvolution (CSD) [9]. All three ODF fields were obtained using: number of reconstruction points (*N*) = 162 angular directions uniformly distributed on a sphere, and diffusion encoding directions (*ND*) = 81 uniform directions on a hemi-sphere. The data used concern two fibers crossing at different angles (90°, 70° 50°, and 30°). The size of the simulated image is 10x10 pixels, and is divided into four regions: the upper left region corresponds to the fibers crossing at 50°, the upper right region at 70°, the lower left region at 90°, and the lower right region at 30°. Each pixel presents two main intravoxel crossing directions.

**Fig 11:**
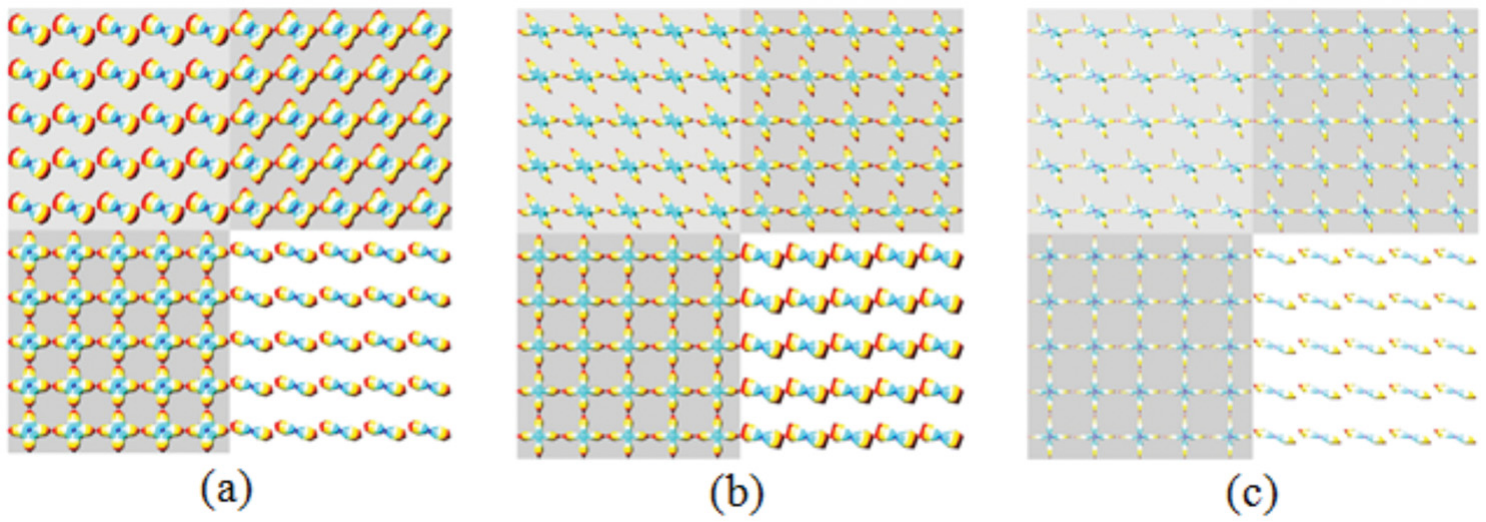
ODFs computed from noise-free diffusion data (b = 5,000 s/mm^2^, *ND* = 81). (a) Field 1. (b) Field 2. (c) Field 3.

To analyze the corresponding 3D point clouds, we used the length ratio, separability, and uncertainty metrics. The experiments were performed in various conditions for the ODF fields: with and without noise, four different crossing angles, and three SNRs. In the noisy cases, we calculated the mean value of the metrics (length ratio, separability, uncertainty), which was obtained by averaging 25 measures over a neighborhood 5×5 pixels, in which the 3D point clouds have the same crossing angle. We illustrate three noise-free ODF fields in Fig 11.

**Fig 12:**
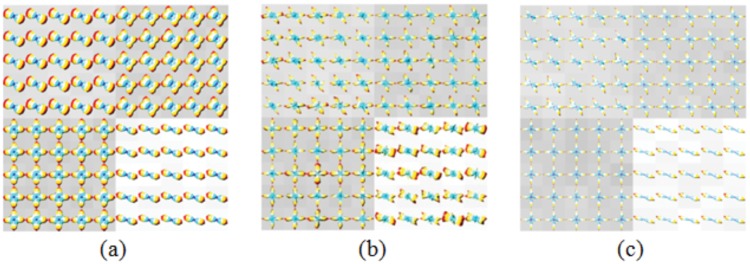
ODFs computed from data having SNR = 20 (b = 5,000 s/mm^2^, *ND* = 81). (a) Field 1. (b) Field 2. (c) Field 3.

We now consider the influence of noise on the characteristic measurement of ODFs in terms of length ratio, separability, and uncertainty, as shown in Fig 12 and Table 2. The ODFs in Fig 12 correspond to SNR = 20. Table 2 gives the results of the quantitative characteristic measurement (mean length ratio, mean separability, and mean uncertainty) of the three ODF fields at different SNRs.

**Table 2:** Characteristic assessment of the three ODF fields at different SNRs, in terms of length ratio, separability, and uncertainty.

Metrics	Crossing angles	SNR = 30			SNR = 20			SNR = 10		
		F1 ^b^	F2 ^b^	F3 ^b^	F1	F2	F3	F1	F2	F3
	90°	0.90	0.90	0.90	0.90	0.85	0.90	0.87	0.80	0.88
**Length ratio**	70°	0.90	0.90	0.90	0.90	0.83	0.90	0.84	0.80	0.85
	50°	0 ^a^	0.86	0.90	0	0.83	0.90	0	0.80	0.83
	30°	0	0.76	0	0	0.61	0	0	0.60	0
	90°	0.5	0.7	0.9	0.5	0.6	0.9	0.4	0.4	0.8
**Separability**	70°	0.2	0.6	0.8	0.2	0.5	0.8	0.2	0.4	0.7
	50°	0	0.5	0.6	0	0.5	0.6	0	0.3	0.6
	30°	0	0.3	0	0	0.3	0	0	0.2	0
	90°	0.26	0.26	0.26	0.26	0.28	0.26	0.28	0.29	0.26
**Uncertainty**	70°	0.30	0.26	0.26	0.30	0.28	0.26	0.37	0.34	0.26
	50°	0.70	0.28	0.26	0.70	0.28	0.26	0.70	0.36	0.28
	30°	0.52	0.35	0.52	0.52	0.37	0.48	0.52	0.37	0.44

^a^ “0” denotes that no peaks are separable in the 3D point cloud.

^b^ F1, F2, and F3 designate Field 1, Field 2, and Field 3, respectively.

In terms of mean length ratio, as the SNR decreased, the length ratios of the three methods were all reduced. The length ratios of Field 2 were more sensitive to SNR in comparison with those of Fields 1 and 3, its length ratios reducing more rapidly as the SNR decreased. The decrease of the length ratios of Field 1 was also greater at smaller crossing angles than at larger angles (90° and 70°), even at SNR = 30; such a decrease is particularly clear compared with noise-free conditions. By contrast, Field 3 showed the most stable behavior.

In terms of separability, Field 1 was less sensitive to noise; its separability only decreased at SNR = 10. However, its peaks were not separable at small crossing angles. The separability of Field 2 reduced more rapidly than the others with the decrease in SNR. Note, however, that the separability at small angles should be interpreted with caution, since the influence of noise on small angle crossings could be too significant as the 3D point clouds are altered by noise. The separability of Field 3 only decreased slightly at SNR = 10.

When the SNR was reduced, the uncertainties were increased. Field 1 presented the greatest uncertainties among these three metrics. The uncertainties of Field 2 were more dependent on noise level than the other two metrics were. The uncertainties of Field 3 were stable.

The results can be summarized as follows. Noise can make the length ratio and separability smaller. Noise can also increase the uncertainty.

For the ODFs (50 in total) containing three fibers crossing each other at 90° in the voxel, the mean length ratio, mean separability, and mean uncertainty are, respectively, 1.9, 1.7, and 1.04 in the noise-free cases. In noisy cases (SNR = 10), the mean length ratio and mean separability decreased to 1.52 and 0.6, respectively, and the mean uncertainty increased to 1.7. The mean separability decreased notably by 1.1 and the uncertainty increased by 0.66 in the noisy cases.

### Results from Real Brain Data

**Fig 13:**
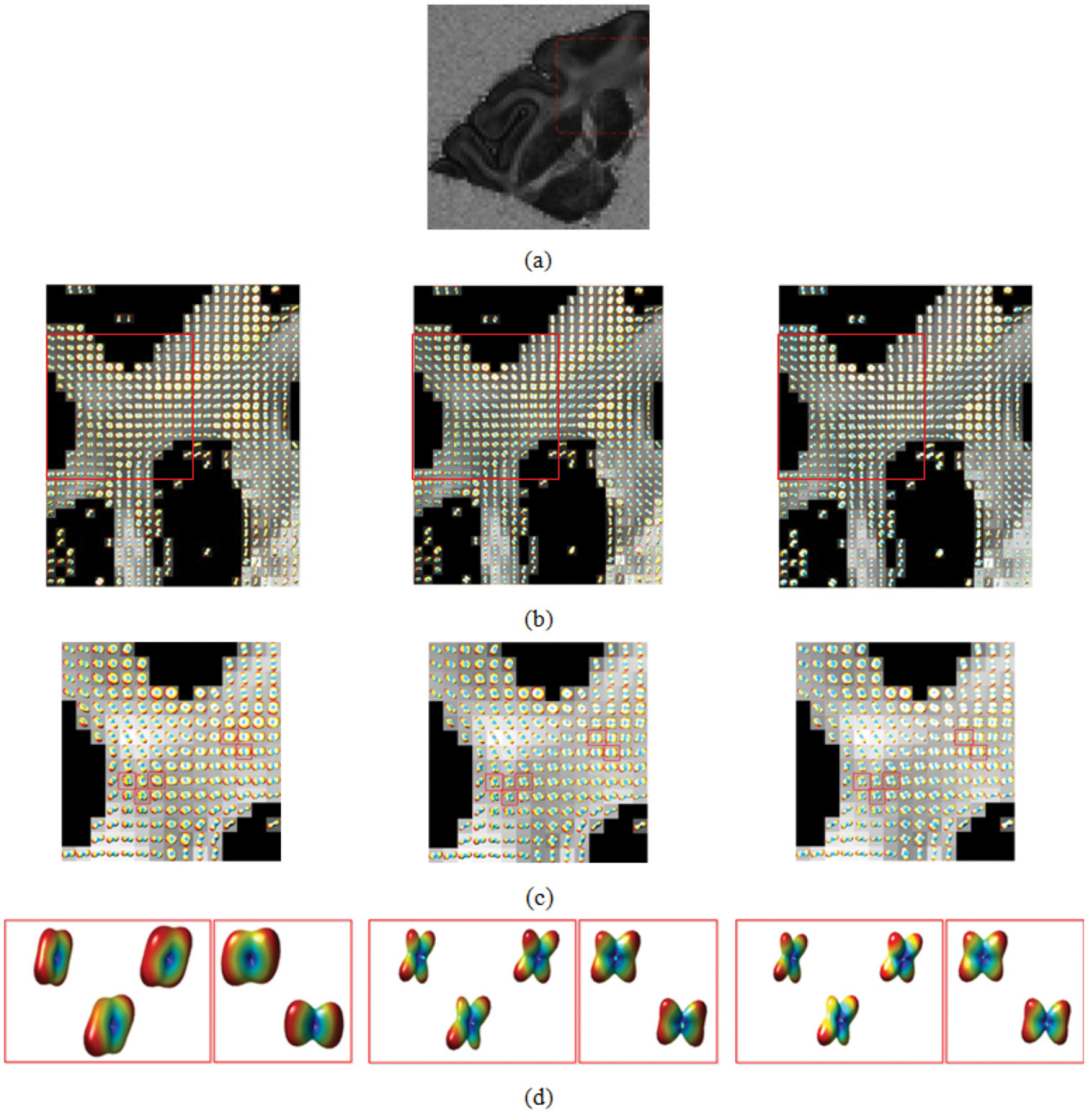
Three ODF fields of real brain data. The ODFs at each voxel are superimposed on a grayscale background modulated by the GFA at that voxel (black: GFA = 0; white: GFA = 1).

In Fig 13(a), we present the GFA map of the brain diffusion-weighted data (black: GFA = 0; white: GFA = 1). Fig 13(b) shows the ODFs (Field 1, Field 2, and Field 3) on a coronal slice at the level of the corpus callosum. Fig 13(c) represents the magnified version of the fiber crossing region circled by the red box in Fig 13(b). Five ODFs corresponding to five different voxels are further magnified in Fig 13(d). The characteristic measurements of the ODFs using the proposed length ratio, separability, and uncertainty are given in Table 3.

**Table 3:** Length ratio, separability, and uncertainty measurements of the three ODF fields in the red box of Fig 13(d).

	Metrics	Voxel 1	Voxel 2	Voxel 3	Voxel 4	Voxel 5
	**Length ratio**	0.90	0.74	0.90	0 ^a^	0
**Field 1**	**Separability**	0.15	0.04	0.10	0 ^a^	0
	**Uncertainty**	0.53	0.70	0.52	1.57	1.22
	**Length ratio**	0.90	0.75	0.95	0.9	0.95
**Field 2**	**Separability**	0.45	0.30	0.45	0.25	0.20
	**Uncertainty**	0.35	0.44	0.26	0.35	0.26
	**Length ratio**	0.85	0.75	0.95	0.85	0.9
**Field 3**	**Separability**	0.40	0.25	0.40	0.20	0.10
	**Uncertainty**	0.44	0.44	0.35	0.44	0.52

^a^”0” denotes that no peaks are separable in the 3D point cloud.

Visually, Field 1 presents poorer separability compared with Fields 2 and 3 and does not allow angular information to be recovered. However, the difference between the ODFs is not always clear visually. This is the case, for example, with Fields 2 and 3 in Fig 13(d). The results in Table 3 give a more quantitative and complete description of the ODFs in Fig 13(d). In terms of length ratio, it is Field 2 that presented the greatest values. In terms of separability, Field 1 presented little separability with a separability value close to null. By contrast, Fields 2 and 3 gave significantly greater (and close) separability values at the same voxel, which implies that they can allow fiber crossing problems to be resolved more easily. In terms of uncertainty, Field 2 also presented the smallest values at the five voxels. At these five voxels, the difference in length ratio, separability, and uncertainty is not very big between Fields 2 and 3, explaining why it is difficult to assess their difference from their surface representations.

Let us consider a region shown in Fig 13(c) (corresponding to the boxed region in Fig 13(b)). Field 1 has a length ratio of nearly 0.9, which means that the lengths of fibers in two directions are nearly the same. Fields 2 and 3 present almost the same length ratio (0.75 for the former and 0.74, respectively). Field 2 has a slightly higher length ratio than Field 3 at nine voxels; the sum of the length ratios in the region is 38.8 for Field 2 and 38.7 for Field 3.

In terms of separability, we found that Field 2 has a higher separability than Field 3 at 18 voxels. The sum of the separability values at all the voxels of the boxed region of Fig 13(c) was equal to 5.15 for Field 2 and 4.45 for Field 3. More precisely, Field 2 has a greater separability than Field 3 at 35.29% (18/51) of the voxels; Field 3 had a greater separability than Field 3 at 11.76% (6/51) of the voxels; for the rest of the voxels, Fields 2 and 3 had the same separability.

In terms of uncertainty, the sum of the uncertainties in the boxed region is 144.9 for Field 1, 113.0 for Field 2, and 114.7 for Field 3. This gives an average uncertainty at a voxel of 0.64 for Field 1, 0.50 for Field 2, and 0.51 for Field 3. If we only sum the voxels at which the fiber crossing is resolved, the average uncertainty obtained is 0.6 for Field 1, 0.52 for Field 2, and 0.53 for Field 3. Therefore, with the uncertainty metric, we can quantitatively say that Fields 2 and 3 resolve fiber crossing more easily than Field 1, and that between Field 2 and Field 3, the former resolves a bit more easily than the latter.

## Discussion

The assessment of the characteristic of ODFs (or any other HARDI quantities that can be taken as 3D point clouds) is challenging to perform due to their shape and topological complexity. Although a few quantitative metrics can be found in the literature, a relatively complete description of ODFs is lacking.

Our results showed that the MSE, sKL, RMSE, and NMSE metrics generated some unreasonable results by ignoring the morphological characteristics of ODFs. With these metrics, irregular ODFs are sometimes taken as better than the ODFs that are in fact obviously closer to the ground truth.

Our results showed that some ODFs (Field 2) can exhibit length ratios greater than the ground truth, which implies that they would have a relatively greater ability to resolve fiber crossing. Field 3 presents smaller length ratios than the ground truth. That is one of the reasons why Field 3 cannot resolve the problem of small angle crossings.

Rician noise can also reduce the separability of ODFs. When the SNR decreases, the separability of ODFs is reduced. We found this to be one of the major difficulties in separating crossing fibers. On the other hand, the separability is lower at low b-values than at high b-values. This conforms to earlier research [28]. The smaller the angle between fibers, the harder it is to distinguish them [3]. Our separability metric enables us to further quantify this. Fields 2 and 3 always present greater separability than Field 1, which explains why they can more easily resolve fiber crossing than Field 1 can.

Rician noise can increase the uncertainty of ODFs. When the SNR decreases, the uncertainty of ODFs increases. Again, this induces another difficulty in separating crossing fibers. When noise increases, uncertainty can increase in an unequal manner in different ODFs. For example, Field 2 underwent more uncertainties than the other two fields, and Field 3 bore smaller uncertainties than Fields 1 and 2. On the other hand, in the case of resolved fiber crossing, the smaller the angle between fibers, the more uncertainty increases with noise. This is the case for Fields 2 and 3, which exhibited smaller uncertainties than Field 1 and therefore resolved fiber crossing more easily.

In the proposed morphological metrics, three important parameters—*N*_*a*_, *N*_*c*_ and *c*_*p*_—are to be determined. The first two parameters can be fixed, depending on the desired accuracy. In the present study, we chose *N*_*a*_ = 9 and *N*_*c*_ = 10 for simulated data and *N*_*a*_ = 9 and *N*_*c*_ = 20 for real data. The choice of *N*_*a*_ = 9 that corresponds to an angular resolution of 10° is based on the fact that the greatest power of resolving fiber crossing is greater than 20°, as indicated in [11,17,29] where the authors resolved fiber crossing with a smallest angle of 28°. When increasing *N*_*a*_, the number of small areas in ADM will increase and the angle interval will be reduced. This will increase the angular precision of the analysis but make the detection of maximum directions more delicate. Likewise, if *N*_*a*_ is too great, the resulting ADM will be too coarse to perform a correct measurement of 3D point clouds. The increase of *N*_*c*_ will increase the number of distance intervals. This can improve the distance precision of analysis, namely, the tiny difference in fiber length ratio as well as in separability, but will also increase computing burden. Furthermore, for most point clouds, *N*_*c*_ = 10 or 20 will be sufficient. If *N*_*c*_ is too small, neighboring points will be mapped in the same set, thus reducing the precision of analysis.

Concerning *c*_*p*_, its setting as 10^−(*i*−1)^ is based on the fact that the nearer the 3D point to the center of sphere, the less it contributes to the shape of 3D point clouds. It is therefore used to weight the role of points as a function of their distance to the center of the sphere.

Concerning reconstruction directions (*N*), its setting as 162 in simulated data or 642 in real data is based on the fact that the reconstruction directions are geometrically uniform directions on a sphere. If *N* is too small, the distances in some directions will not be calculated. Reducing *N* will increase angular error and make some rows of ADMAT be zero. However, increasing *N* will increase the number of 3D points, and this will make the ADMAT more accurate, but will increase computing burden. Usually, *N* = 642 is sufficient.

Nonetheless, several limitations of the metrics proposed here merit discussion:

Firstly, projecting 3D point clouds in 2D ADMAT enables us to readily compute morphological metrics. However, in doing so, we lose rotation information in 3D space, and the projection will affect the estimation of the angle of a fiber with respect to a fixed reference axis (positive x-axis, for example). The advantage is, however, that the proposed morphological metrics are rotationally invariant. This is all the more true as we are not focusing on the measurement of relative angles between two fibers. Secondly, we need to choose a reference direction (the direction of the vector having the maximal radial distance). It is possible that there is more than one maximal radial distance point in the 3D point cloud. In this case, we can select any of them as reference direction. This is because the proposed morphological metrics do not change with reference direction. However, the estimation of the angle of a fiber with respect to a given reference axis will change if a different direction is used as the reference direction.

## Conclusions

We have proposed a novel paradigm allowing the characteristics of general 3D point clouds including the ODF in HARDI to be assessed. The paradigm is based on the measurement of the morphological characteristics of 3D point clouds. In this framework, three particular quantitative metrics have been proposed. The results showed that the proposed morphological metrics are consistent with the visual quality of 3D point clouds and enable us to describe quantitatively and accurately the characteristics of the latter, which provides a new way of quantifying the characteristics and potentially the quality of 3D point clouds in resolving fiber crossings.

## Supporting Information

S1 Dataset The brain data used in this research. (MAT)

## References

[1] Wedeen VJV, Hagmann P, Tseng W-YI, Reese TG, Weisskoff RM (2005) Mapping complex tissue architecture with diffusion spectrum magnetic resonance imaging. Magn Reson Med 54: 1377–1386. 16247738 10.1002/mrm.20642

[2] Tuch DS (2004) Q-ball imaging. Magn Reson Med 52: 1358–1372. 15562495 10.1002/mrm.20279

[3] Descoteaux M, Angelino E, Fitzgibbons S, Deriche R (2007) Regularized, fast, and robust analytical Q-ball imaging. Magn Reson Med 58: 497–510. 17763358 10.1002/mrm.21277

[4] Michailovich O, Rathi Y, Shenton ME (2010) On Approximation of Orientation Distributions by means of Spherical Ridgelets. IEEE Trans image Process 19: 461–477. 10.1109/TIP.2009.2035886 19887312 PMC3073602

[5] Hess CP, Mukherjee P, Han ET, Xu D, Vigneron DB (2006) Q-ball reconstruction of multimodal fiber orientations using the spherical harmonic basis. Magn Reson Med 56: 104–117. 16755539 10.1002/mrm.20931

[6] Kezele I, Descoteaux M, Poupon C, Poupon F, Mangin JF (2010) Spherical wavelet transform for ODF sharpening. Med Image Anal 14: 332–342. 10.1016/j.media.2010.01.002 20207188

[7] Descoteaux M, Deriche R, Knösche TR, Anwander A (2009) Deterministic and probabilistic tractography based on complex fibre orientation distributions. IEEE Trans Med Imaging 28: 269–286. 10.1109/TMI.2008.2004424 19188114

[8] Michailovich O, Rathi Y, Dolui S (2011) Spatially Regularized Compressed Sensing for High Angular Resolution Diffusion Imaging. IEEE Trans Med Imaging 30: 1100–1115. 10.1109/TMI.2011.2142189 21536524 PMC3708319

[9] Tournier J-D, Yeh C-H, Calamante F, Cho K-H, Connelly A, Lin CP. (2008) Resolving crossing fibres using constrained spherical deconvolution: validation using diffusion-weighted imaging phantom data. Neuroimage 42: 617–625. 10.1016/j.neuroimage.2008.05.002 18583153

[10] Tournier J-D, Calamante F, Gadian DG, Connelly A (2004) Direct estimation of the fiber orientation density function from diffusion-weighted MRI data using spherical deconvolution. Neuroimage 23: 1176–1185. 15528117 10.1016/j.neuroimage.2004.07.037

[11] Özarslan E, Shepherd T, Vemuri B (2006) Resolution of complex tissue microarchitecture using the diffusion orientation transform (DOT). Neuroimage 31: 1086–1103. 16546404 10.1016/j.neuroimage.2006.01.024

[12] Jian B, Vemuri BC, Ozarslan E, Carney PR, Mareci TH (2007) A novel tensor distribution model for the diffusion-weighted MR signal. Neuroimage 37: 164–176. 17570683 10.1016/j.neuroimage.2007.03.074 PMC2576290

[13] Daducci A, Canales-Rodríguez EJ, Descoteaux M, Garyfallidis E, Gur Y, Lin YC et al (2014) Quantitative comparison of reconstruction methods for intra-voxel fiber recovery from diffusion MRI. IEEE Trans Med Imaging 33: 384–399. 10.1109/TMI.2013.2285500 24132007

[14] Dolui S, Michailovich OV, Rathi Y (2011) Compressed sensing of diffusion MRI data using spatial regularization and positivity constraints IEEE International Symposium on Biomedical Imaging : From Nano to Macro. pp. 1597–1601.

[15] Ozarslan E, Vemuri BC, Mareci TH (2005) Generalized scalar measures for diffusion MRI using trace, variance, and entropy. Magn Reson Med 53: 866–876. 15799039 10.1002/mrm.20411

[16] Zhan L, Leow AD, Barysheva M, Feng A, Toga AW, Sapiro G, et al (2009) Investigating the uncertainty in multi-fiber estimation in high angular resolution diffusion imaging Med. Image Comput. Comput. Assist. Interv. (MICCAI). Springer Berlin Heidelberg pp. 256–267.

[17] Aganj I, Lenglet C, Sapiro G, Yacoub E, Ugurbil K, Harel N. (2010) Reconstruction of the Orientation Distribution Function in Single and Multiple Shell Q-Ball Imaging within Constant Solid Angle. Magn Reson Med 64: 554–566. 10.1002/mrm.22365 20535807 PMC2911516

[18] Hartigan JAJ, Hartigan PMP (1985) The dip test of unimodality. Ann Stat 13: 70–84.

[19] Seunarine KK, Cook P a, Hall MG, Embleton K V, Parker GJM, Alexander D C. (2007) Exploiting peak anisotropy for tracking through complex structures. IEEE 11th Int Conf Comput Vis: 1–8.

[20] Cohen-Adad J, Descoteaux M, Wald LL (2011) Quality assessment of high angular resolution diffusion imaging data using bootstrap on Q-ball reconstruction. J Magn Reson Imaging 33: 1194–1208. 10.1002/jmri.22535 21509879 PMC3087444

[21] Schwab E, Cetingül HE, Afsari B, Vidal R (2013) Rotation invariant features for HARDI. Inf Process Med Imaging 23: 705–717. 24684011 10.1007/978-3-642-38868-2_59 PMC4194072

[22] Chu CY, Huang JP, Sun CY, Liu WY, Zhu YM (2015) Resolving intravoxel fiber architecture using nonconvex regularized blind compressed sensing. Phys Med Biol.60(6):2339–2354. 10.1088/0031-9155/60/6/2339 25716031

[23] Sun CY, Chu CY, Liu WY, Hsu EW, Korenberg JR, Zhu YM (2015) Quantitative representation and description of intravoxel fiber complexity in HARDI. Phys Med Biol. 60(21):8417–8436. 10.1088/0031-9155/60/21/8417 26464329

[24] Reisert M, Kiselev VG (2011) Fiber continuity: an anisotropic prior for ODF estimation. IEEE Trans Med Imaging 30: 1274–1283. 10.1109/TMI.2011.2112769 21317082

[25] Welsh CL, DiBella EVR, Adluru G, Hsu EW (2013) Model-based reconstruction of undersampled diffusion tensor k-space data. Magn Reson Med 70: 429–440. 10.1002/mrm.24486 23023738 PMC4469271

[26] Tuch DS, Reese TG, Wiegell MR, Makris N, Belliveau JW, Wedeen VJ (2002) High angular resolution diffusion imaging reveals intravoxel white matter fiber heterogeneity. Magn Reson Med 48: 577–582. 12353272 10.1002/mrm.10268

[27] Kingsley PB (2006) Introduction to diffusion tensor imaging mathematics: Part I. Tensors, rotations, and eigenvectors. Concepts Magn Reson Part A 28A: 101–122.

[28] Frank LR (2001) Anisotropy in high angular resolution diffusion-weighted MRI. Magn Reson Med 45: 935–939. 11378869 10.1002/mrm.1125

[29] Jian B, Vemuri BC, Özarslan E (2009) A Mixture of Wisharts (MOW) Model for Multifiber Reconstruction In: Laidlaw D, Weickert J, editors. Visualization and Processing of Tensor Fields. Mathematics and Visualization. Springer Berlin Heidelberg pp. 39–56.

